# Trends in Cardiovascular Disease Risk Factor Prevalence and Estimated 10-Year Cardiovascular Risk Scores in a Large Untreated French Urban Population: The CARVAR 92 Study

**DOI:** 10.1371/journal.pone.0124817

**Published:** 2015-04-23

**Authors:** Carma Karam, Alain Beauchet, Sebastien Czernichow, Florence de Roquefeuil, Alain Bourez, Nicolas Mansencal, Olivier Dubourg

**Affiliations:** 1 Cardiology Department, Hôpital Ambroise Paré, Assistance Publique-Hôpitaux de Paris (AP-HP), Centre de référence des Maladies Cardiaques Héréditaires, Université de Versailles-Saint Quentin (UVSQ), Boulogne-Billancourt, France; 2 Public Health Department, Hôpital Ambroise Paré, AP-HP, UVSQ, Boulogne-Billancourt, France; 3 Nutrition Department, Hôpital Ambroise Paré, AP-HP, UVSQ, Boulogne-Billancourt, France; 4 INSERM UMS-011, Population-Based Epidemiological Cohorts, Villejuif, France; 5 Local Health Insurance, Managing Director, Nanterre, France; 6 INSERM U-1018, CESP, Team 5 (EpReC, Renal and Cardiovascular Epidemiology), UVSQ, Villejuif, France; National University of Singapore, SINGAPORE

## Abstract

**Background:**

Surveys measuring effectiveness of public awareness campaigns in reducing cardiovascular disease (CVD) incidence have yielded equivocal findings. The aim of this study was to describe cardiovascular risk factors (CVRFs) changes over the years in an untreated population-based study.

**Methods:**

Between 2007 and 2012, we conducted a screening campaign for CVRFs in men aged 40 to 65 yrs and women aged 50 to 70 yrs in the western suburbs of Paris. Data were complete for 20,324 participants of which 14,709 were untreated.

**Results:**

The prevalence trend over six years was statistically significant for hypertension in men from 25.9% in 2007 to 21.1% in 2012 (p=0.002) and from 23% in 2007 to 12.7% in 2012 in women (p<0.0001). The prevalence trend of tobacco smoking decreased from 38.6% to 27.7% in men (p=0.0001) and from 22.6% to 16.8% in women (p=0.113). The Framingham 10-year risk for CVD decreased from 13.3 ± 8.2 % in 2007 to 11.7 ± 9.0 % in 2012 in men and from 8.0 ± 4.1 % to 5.9 ± 3.4 % in women. The 10-year risk of fatal CVD based on the European Systematic COronary Risk Evaluation (SCORE) decreased in men and in women (p <0.0001).

**Conclusions:**

Over a 6-year period, several CVRFs have decreased in our screening campaign, leading to decrease in the 10-year risk for CVD and the 10-year risk of fatal CVD. Cardiologists should recognize the importance of community prevention programs and communication policies, particularly tobacco control and healthier diets to decrease the CVRFs in the general population.

## Introduction

Ischemic heart disease is the leading cause of death worldwide [1], followed by stroke and other cerebrovascular diseases. Cardiovascular disease (CVD) is still in the top two causes of mortality in France[2] with a rate of 237/100,000 in 2006.

The major cardiovascular risk factors (CVRFs) are known: diabetes, hypertension, dyslipidemia and smoking are associated with an increased risk of coronary heart disease [3–8]. Controlling these factors has been shown to help reduce the CVD risk level[9]. Thus, CVRF screening may detect these modifiable factors early and thereby improve patients’ life expectancy and functional status.

Public campaigns seek to raise awareness and focus on prevention and education in terms of physical activity [10–12] and healthy and balanced diets[13–18], and anti-tobacco legislation has banned smoking in closed public spaces. However, surveys measuring effectiveness in reducing CVD incidence have yielded equivocal findings[10–12]. Since February 2007, French law requires food brands to include health messages in advertisements for food and beverages on television and radio and in newspapers. Products affected by this measure are foods and drinks containing added sugar, salt or sweeteners, and processed foods. A ban on smoking in all public places was implemented in France in 2007.

The cardiovascular (CV) division of our university hospital in the western suburbs of Paris, jointly with the local health insurance body has been running a CVRF screening program since 2007: the CARVAR (CARdioVAscular Risk factors) 92 study. Our purpose was to describe the CVRF changes over the years in the untreated participants of this large population-based study.

## Material and Methods

### Population

Between January 2007 and December 2012, we conducted a screening campaign in the western suburbs of Paris (the CARVAR 92 study). The target population was men aged 40 to 65 years and women aged 50 to 70 years. The social insured inhabitants of the western suburbs of Paris matching the age and sex requirements were sent a form inviting them to a free medical visit in one of the participating centers. They were asked about their personal and family history of CVD, and whether they were taking any medication. To determine current cigarette smoking, respondents were asked if they smoked at least one cigarette per day for at least 6 months over the last three years. Weight and height were measured by a standard protocol and used to calculate body mass index (BMI); i.e, weight in kilograms divided by the square of height in meters. Systolic and diastolic blood pressure (BP) was measured according to standard protocols in a supine position. Screening included blood tests for total cholesterol, low-density lipoprotein-cholesterol (LDL-C), high-density lipoprotein-cholesterol (HDL-C), triglycerides and glucose with 12 hours of fasting prior to the blood draw using standardized methods. During the examination and face-to-face interview, physicians completed an online questionnaire including information about previous and discovered CVRFs and the results of the blood tests. The software (http://www.cpam92-si.com/site/frcv/frcv.php) calculated the participants’ 10-year risk for CVD (coronary, cerebrovascular, and peripheral arterial disease and heart failure) using the d’Agostino-method[19] and the European Systematic COronary Risk Evaluation (SCORE) [20] estimation of 10-year risk of fatal CVD. The access to the website was freely available to calculate the 10-year risk scores, but recording of data was protected by access codes. It was used for educational/information purposes, reinforced with possible simulation in the presence of the person at risk. Printed results were given to the participants and sent to their general practitioner (GP). High-risk patients were offered further care at the university hospital while low- and medium-risk patients were advised to visit their GP. An interview with a nutritionist and a smoking cessation specialist were offered to all study participants, who were encouraged to answer a satisfaction survey. The study was approved by the National Commission for Data Protection and Liberties (CNIL-France). The Comité de Protection des Personnes reviewed the study and provided a formal statement declaring this study to be exempt from the requirement for human research ethics approval.

### Cardiovascular Risk Factors and 10-Year Risk for CVD

Diabetes mellitus was defined as fasting plasma glucose value ≥7 mmol/L, hypertension as BP exceeding 140 over 90 mmHg in nondiabetics and 130 over 80 mmHg in diabetic patients, obesity as a BMI ≥30 kg/m^2^ and high LDL-Cas a fasting plasma value ≥4.14 mmol/L[21–23]. Current smoking was defined as a positive answer to the question above. Subjects who stopped smoking for at least 3 years were considered non-smokers. For the Framingham 10-year risk for CVD, low risk was defined as < 10%, intermediate risk as 10% to 20%, and high risk as > 20%, while for the European 10-year risk of fatal CVD, low risk was defined as < 2%, intermediate risk as ≥ 2% and < 5%, and high risk as ≥ 5%.

### Statistical Analysis

Three populations were defined. Population A consisted of the total participants who presented to the medical visit and for whom data were complete. Population B consisted of the total participants who presented to the medical visit and for whom data were complete and who were not taking any antihypertensive or lipid-lowering agents or drug treatment for diabetes. Since the screening program addressed older people with social insurance in the early years and younger ones in the later years, we adjusted the results according to gender and ± 5-year age groups for the study participants who were not taking any antihypertensive or lipid-lowering agents or drug treatment for diabetes. Population C represented the age- and sex-adjusted untreated population. We used direct methods for adjustment and matched each study subject in 2007 by sex and age (± 5 years) with other study subjects in 2008, 2009, 2010, 2011 and 2012 successively. Quantitative data are expressed as mean ± standard deviation and qualitative data as frequency and percent. Comparisons of means were performed using the Student t test and Analysis of Variance. Linear trends were verified using the Cochran-Armitage trend test for linearity for categorical data (diabetes, hypertension, high LDL-C, current smokers, obesity), and regression lines for parametric data (10-year risk of fatal CVD and 10-year risk of CVD). A p value less than .05 was considered statistically significant. All statistical analyses were performed with the use of SAS statistical software (version 9.3, SAS Institute Inc., Cary, North Carolina, USA).

## Results

### Populations Characteristics

**Fig 1:**
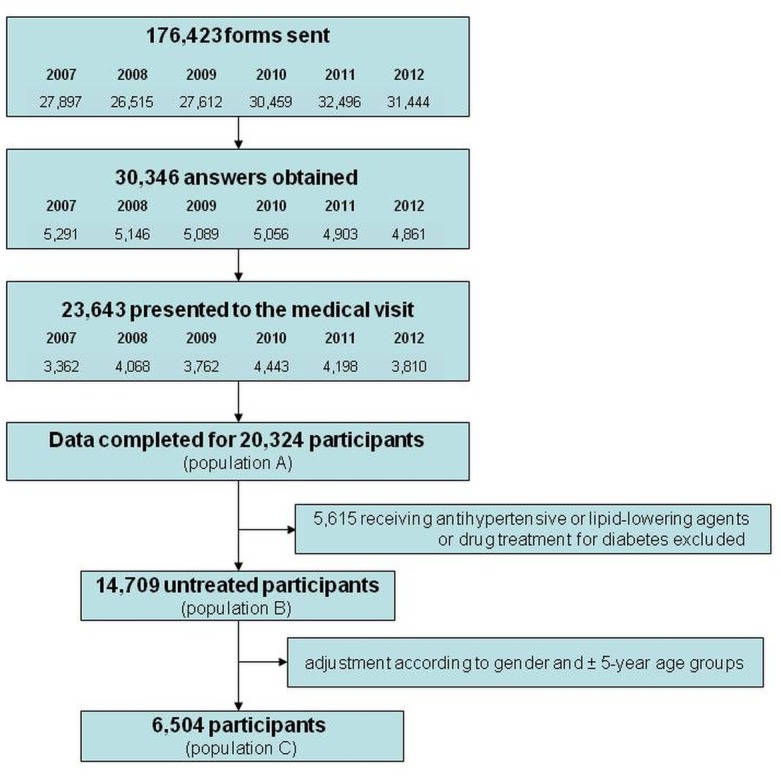
Selection of the CARVAR 92 study populations.

On December 31, 2012; 177,000 (51%) of the 347,396 inhabitants of the western suburbs of Paris with social insurance matching the age and sex requirements had already received a form inviting them to a free medical visit in one of the participating centers. A total of 30,646 answers were obtained and 23,643 social insured presented to the medical visit. Data were complete for 20,324 participants (S1 Table) of whom 14,709 did not receive any antihypertensive or lipid-lowering agents or drug treatment for diabetes. Fig 1 shows the selection of the study populations (flow chart). The characteristics of population A are summarized in Table 1 and the characteristics of population B are shown in Table 2. In population A, the sex ratio (male/female) was 0.89 and the mean age was 51.5 ± 7.9 years in men and 58.1 ± 7.2 years in women. Hypertension was found to be the most common CVRF (34.6%) followed by high LDL-C (34.4%), current smoking (20.7%), and obesity (18%). Diabetes was found in 8.7% of the total population. In population B, the sex ratio (male/female) was 1.01 and the mean age was 50.1 ± 7.7 years in men and 56.9 ± 7.1 years in women. High LDL-C was found to be the most common CVRF (24.3%) followed by current smoking (22.5%), hypertension (17.4%) and obesity (13.4%). Diabetes was found in only 2.1% of the untreated participants.

**Table 1:** Characteristics of the participants in population A.

		Total	Women	Men
		n = 20,324	n = 10,740	n = 9,584
Age (years)		55.0 ± 8.2	58.1 ± 7.2	51.5 ± 7.9
Mean systolic BP (mm Hg)		126.3 ± 15.1	125.0 ± 15.5	127.7 ± 14.4
Mean diastolic BP (mm Hg)		76.9 ± 9.5	75.9 ± 9.3	78.1 ± 9.6
Fasting total cholesterol (mmol/L)		5.59 ± 1.06	5.70 ± 1.09	5.49 ± 1.01
Fasting LDL cholesterol (mmol/L)		3.47 ± 0.96	3.47 ± 0.96	3.50 ± 0.96
Fasting HDL cholesterol (mmol/L)		1.55 ± 0.54	1.71 ± 0.57	1.35 ± 0.44
Fasting triglycerides (mmol/L)		1.29 ± 0.84	1.15 ± 0.63	1.44 ± 0.99
BMI (kg/m^2^)		26.16 ± 4.63	26.14 ± 5.23	26.19 ± 3.85
Fasting plasma glucose (mmol/L)		5.55 ± 1.22	5.44 ± 1.17	5.61 ± 1.33
Diabetes mellitus, n (%)		1,771 (8.7)	897 (8.3)	874 (9.1)
Hypertension, n (%)		7,022 (34.6)	3,796 (35.3)	3,226 (33.7)
High LDL cholesterol, n (%)		7,000 (34.4)	3,841 (35.7)	3,159 (33.0)
Obesity, n (%)		3,662 (18.0)	2,247 (20.9)	1,415 (14.7)
Current smokers, n (%)		4,206 (20.7)	1,655 (15.4)	2,551 (26.6)
10-year risk for CVD (%)		11.2 ± 9.03	8.49 ± 6.55	14.07 ± 10.41
10-year risk of fatal CVD (%)		1.07 ± 1.16	1.01 ± 1.16	1.15 ± 1.16
10-year risk for CVD				
	<10%	11,971 (58.9%)	7,851 (73.1%)	4,121 (43.0%)
	10–20%	5,772 (28.4%)	2,266 (21.1%)	3,498 (36.5%)
	>20%	2,581 (12.7%)	623 (5.8%)	1,965 (20.5%)
10-year risk of fatal CVD				
	< 2%	17,276 (85.0%)	9,215 (85.8%)	8,060 (84.1%)
	≥ 2% < 5%	2,845 (14.0%)	1,450 (13.5%)	1,390 (14.5%)
	≥ 5%	203 (1.0%)	75 (0.7%)	134 (1.4%)

Population A consisted of the total participants who presented to the medical visit and for whom data were complete.

**Table 2:** Characteristics of the untreated participants (population B).

		Total	Women	Men
		n = 14,709	n = 7,308	n = 7,401
Age (years)		53.5 ± 8.2	56.9 ± 7.1	50.1 ± 7.7
Mean systolic BP (mm Hg)		124.3 ± 14.2	122.1 ± 14.2	126.3 ± 13.9
Mean diastolic BP (mm Hg)		76.3 ± 9.3	75.0 ± 9.0	77.6 ± 9.47
Fasting total cholesterol (mmol/L)		5.67 ± 0.98	5.80 ± 0.96	5.57 ± 0.98
Fasting LDL cholesterol (mmol/L)		3.55 ± 0.93	3.55 ± 0.96	3.57 ± 0.91
Fasting HDL cholesterol (mmol/L)		1.55 ± 0.49	1.76 ± 0.47	1.37 ± 0.44
Fasting triglycerides (mmol/L)		1.23 ± 0.78	1.07 ± 0.58	1.39 ± 0.90
BMI (kg/m^2^)		25.5 ± 4.2	25.2 ± 4.8	25.7 ± 3.6
Fasting plasma glucose (mmol/L)		5.33 ± 0.83	5.22 ± 0.78	5.44 ± 0.89
Diabetes mellitus, n (%)		318 (2.1)	123 (1.7)	195 (2.6)
High LDL cholesterol, n (%)		3,576 (24.3)	1,748 (14.5)	1,828 (20.3)
Hypertension, n (%)		2,564 (17.4)	1,062 (14.5)	1,502 (20.3)
Obesity, n (%)		1,971(13.4)	1,114 (15.2)	857 (11.6)
Current smokers, n (%)		3,312 (22.5)	1,240 (17.0)	2,072 (28.0)
10-year risk for CVD (%)		9.15 ± 7.07	6.53 ± 4.30	11.74 ± 8.2
10-year risk of fatal CVD (%)		0.91 ± 0.99	0.82 ± 0.88	0.99 ± 1.07
10-year risk for CVD				
	<10%	10,062 (68.4%)	6,236 (85.3%)	3,826 (51.7%)
	10–20%	3,592 (24.4%)	967 (13.2%)	2,625 (35.5%)
	>20%	1,055 (7.2%)	105 (1.5%)	950 (12.8%)
10-year risk of fatal CVD				
	< 2%	13,093 (89.0%)	6,591 (90.2%)	6,505 (87.8%)
	≥ 2% < 5%	1,512 (10.3%)	685 (9.4%)	827 (11.2%)
	≥ 5%	104 (0.7%)	32 (0.4%)	72 (1.0%)

Population B consisted of the total participants who presented to the medical visit and for whom data were complete and who were not taking any antihypertensive or lipid-lowering agents or drug treatment for diabetes

There were no statistically significant differences between population B and population C (adjusted model) concerning the distribution of the CVRFs (S2 Table).

### Risk Factor Prevalence and 10-Year Risk for CVD in the Untreated Participants

In population B, 42.5% were found to be free of all major CVRFs, 56.8% had between 1 and 3 CVRFs and only 0.7% had four or more CVRFs. The predicted 10-year risk for CVD ranked men significantly more at high risk than women (12.8% vs 1.5%, p <0.0001). Similarly, according to the European SCORE estimation of the 10-year risk of fatal CVD, the prevalence of high-risk individuals was significantly higher in men than in women (1.0% vs 0.4%, p <0.0001).

### CVRF Changes Between 2007 and 2012

**Fig 2:**
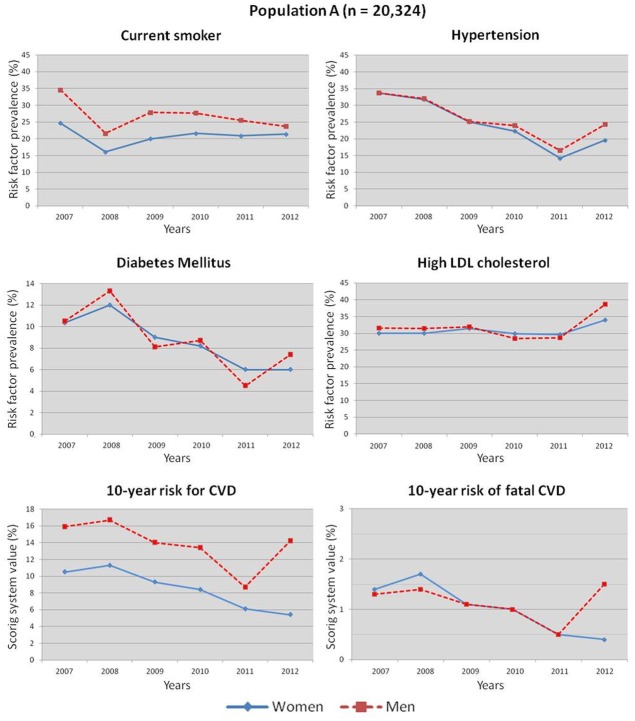
Cardiovascular risk factors and estimated 10-year risk for CVD and fatal CVD between 2007 and 2012 in population A.

**Fig 3:**
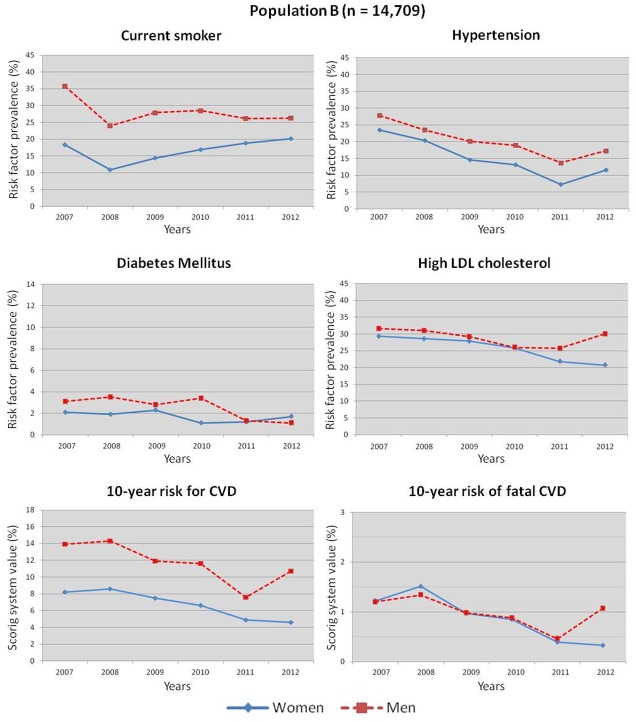
Cardiovascular risk factors and estimated 10-year risk for CVD and fatal CVD between 2007 and 2012 in population B.

**Fig 4:**
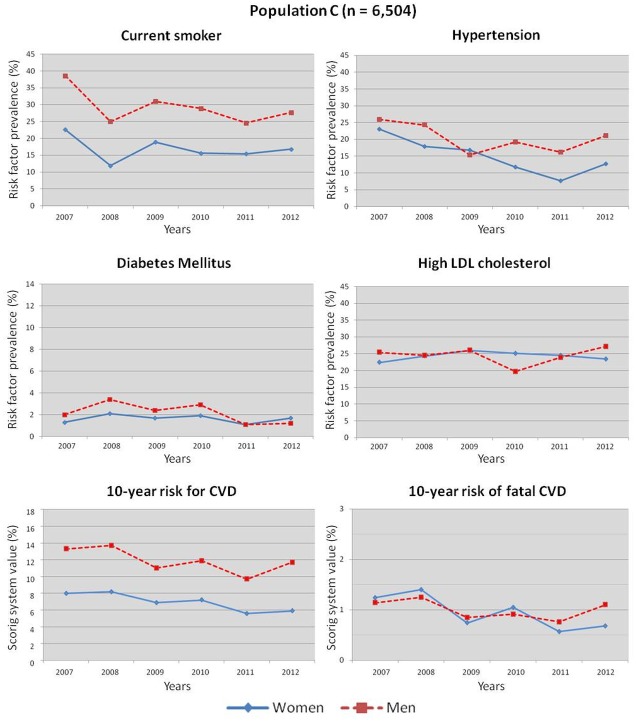
Cardiovascular risk factors and estimated 10-year risk for CVD and fatal CVD between 2007 and 2012 (adjusted model).

Changes in CVRFs and the 10-year risk scoring systems between 2007 and 2012 are represented in Fig 2 and S3 Table for population A, in Table 3 and Fig 3 for population B and Table 4 and Fig 4 for the adjusted model (population C). Fig 2 shows the line graphs of the changes in CVRFs and the 10-year risk scoring systems in population A between 2007 and 2012. In the male population, we observed a decrease in the prevalence of all CVRFs and in both 10-year risk for CVD and fatal CVD scores. In women, all but high LDL-C prevalence decreased. Fig 3 shows the line graphs of the changes in CVRFs and the 10-year risk scoring systems in population B between 2007 and 2012. In the male population, we observed a decrease in the prevalence of all CVRFs and in both 10-year risk for CVD and fatal CVD scores. In women, all but obesity and diabetes prevalence decreased.

**Table 3:** Cardiovascular risk factors and estimated 10-year risk for CVD and fatal CVD in population B (N = 14,709).

Men	2007	2008	2009	2010	2011	2012	P value
N = 7,401	1,160	1,398	1,116	1,510	1,142	1,075	
Hypertension (%)	27.8	23.5	20.1	18.9	13.7	17.3	<0.0001
Diabetes mellitus (%)	3.1	3.5	2.8	3.4	1.3	1.1	<0.0001
High LDL-C (%)	31.6	31.0	29.2	26.0	25.7	30.0	0.009
Obesity (%)	14.7	12.0	10.6	10.1	10.4	12.0	0.014
Current smokers (%)	35.8	24.0	27.9	28.5	26.1	26.3	0.001
10-year risk for CVD (%) *	13.9 ± 8.2	14.3 ± 8.3	11.9 ± 7.5	11.6 ± 7.8	7.6 ± 5.7	10.7 ± 9.5	<0.0001
10-year risk of fatal CVD (%)*	1.20 ± 0.91	1.34 ± 0.98	0.98 ± 0.96	0.88 ± 0.82	0.46 ± 0.66	1.07 ± 1.70	<0.0001
**Women**	**2007**	**2008**	**2009**	**2010**	**2011**	**2012**	**P value**
N = 7,308	1,215	950	1,156	1,111	1,552	1,324	
Hypertension (%)	23.5	20.4	14.6	13.2	7.3	11.6	<0.0001
Diabetes mellitus (%)	2.1	1.9	2.3	1.1	1.2	1.7	0.052
High LDL-C (%)	29.3	28.6	27.9	25.9	21.8	20.7	<0.0001
Obesity (%)	16.9	14.8	13.8	15.1	14.2	16.5	0.753
Current smokers (%)	18.3	10.9	14.4	16.9	18.8	20.2	<0.0001
10-year risk for CVD (%)*	8.2 ± 4.8	8.6 ± 4.7	7.5 ± 4.7	6.6 ± 4.0	4.9 ± 3.0	4.6 ± 3.0	<0.0001
10-year risk of fatal CVD (%)*	1.22 ± 0.93	1.51 ± 1.08	0.97 ± 0.92	0.85 ± 0.78	0.39 ± 0.40	0.3 3± 0.49	<0.0001

*Mean ± SD

Population B consisted of the total participants who presented to the medical visit and for whom data were complete and who were not taking any antihypertensive or lipid-lowering agents or drug treatment for diabetes

Linear trends were verified using the Cochran-Armitage trend test for linearity for categorical data (diabetes, hypertension, high LDL-C, obesity, current smokers), and regression lines for parametric data (10-year risk of fatal CVD and 10-year risk of CVD)

CVD = cardiovascular disease; LDL-C = low-density lipoprotein-cholesterol

**Table 4:** Cardiovascular risk factors and estimated 10-year risk for CVD and fatal CVD in adjusted model (population C) (N = 6,504).

Men	2007	2008	2009	2010	2011	2012	P value
N = 3,402	567	567	567	567	567	567	
Hypertension (%)	25.9	24.3	15.4	19.2	16.2	21.1	0.002
Diabetes mellitus (%)	2.0	3.4	2.4	2.9	1.1	1.2	0.035
High LDL-C (%)	25.4	24.5	26.0	19.7	23.9	27.1	0.206
Obesity (%)	14.9	13.5	11.8	11.1	10.2	11.6	0.023
Current smokers (%)	38.6	25.0	31.0	28.9	24.6	27.7	0.001
10-year risk for CVD (%) *	13.3 ± 8.2	13.7 ± 7.6	11.0 ± 7.9	11.9 ± 7.1	9.7 ± 6.6	11.7 ± 9.0	<0.0001
10-year risk of fatal CVD (%)*	1.14 ± 1.01	1.25 ± 0.88	0.85 ± 0.97	0.91 ± 0.63	0.76 ± 0.80	1.10 ± 1.58	<0.0001
**Women**	**2007**	**2008**	**2009**	**2010**	**2011**	**2012**	**P value**
N = 3,102	517	517	517	517	517	517	
Hypertension (%)	23.0	17.9	16.8	11.8	7.7	12.7	<0.0001
Diabetes mellitus (%)	1.3	2.1	1.7	1.9	1.1	1.7	0.87
High LDL-C (%)	22.4	24.3	25.9	25.1	24.5	23.4	0.542
Obesity (%)	17.0	14.1	11.8	13.7	17.9	15.2	0.713
Current smokers (%)	22.6	11.9	18.9	15.6	15.4	16.8	0.113
10-year risk for CVD (%)*	8.0 ± 4.1	8.2 ± 4.3	6.9 ± 4.4	7.2 ± 4.4	5.6 ± 3.0	5.9 ± 3.4	<0.0001
10-year risk of fatal CVD (%)*	1.24 ± 1.12	1.40 ± 0.97	0.74 ± 0.81	1.05 ± 0.88	0.57 ± 0.53	0.68 ± 0.73	<0.0001

*Mean ± SD

Population C represented the age- and sex-adjusted untreated population.

Linear trends were verified using the Cochran-Armitage trend test for linearity for categorical data (diabetes, hypertension, high LDL-C, obesity, current smokers), and regression lines for parametric data (10-year risk of fatal CVD and 10-year risk of CVD).

CVD = cardiovascular disease; LDL-C = low-density lipoprotein-cholesterol

In the adjusted model (Fig 4), the prevalence trend over the six years of the study was statistically significant for hypertension in men from 25.9% in 2007 to 21.1% in 2012 (trend test, p = 0.002) and from 23% in 2007 to 12.7% in 2012 in women (trend test, p <0.0001). Similarly, the prevalence trend of current tobacco smoking decreased significantly from 38.6% in 2007 to 27.7% in 2012 in men (trend test, p = 0.0001). It decreased from 22.6% in 2007 to 16.8% in 2012 in women, but the trend over the six years of the study was not statistically significant (trend test, p = 0.113).

We observed a significant decrease in the mean 10-year risk for CVD from 13.3 ± 8.2% in 2007 to 11.7 ± 9.0% in 2012 in men and from 8.0 ± 4.1% in 2007 to 5.9 ± 3.4% in women (both p <0.0001). The 10-year risk of fatal CVD showed a significant decrease in men and in women (1.2 ± 1.1% in 2007 and 0.6 ± 0.7% in 2012, p <0.0001). In the male population, we observed a significant decrease in obesity and diabetes mellitus but not in high LDL-C. Moreover, high LDL-C, diabetes and obesity did not vary significantly in women.

### Satisfaction Survey Statements

Responses to statements regarding to the study showed a high level of agreement with the need for screening. The great majority of service users (92%) had a positive experience of the screening service, agreeing that they were given enough time and attention.

## Discussion

This is the first large multiple cross-sectional population-based study to address the prevalence of the main CVRFs and 10-year risk for CVD scoring systems in the western suburbs of Paris. In 2007, the French legislation banned smoking in public spaces and required food brands to include health messages in advertisements on television, radio and newspapers. Our purpose was to describe the changes in CVRFs between 2007 and 2012 in the untreated population, i.e. the study participants who were not taking any antihypertensive or lipid-lowering agents or drug treatment for diabetes.

In the male population, we observed a significant decrease in the prevalence trend of hypertension, tobacco smoking and diabetes as well as in the 10-year risk for CVD and the 10-year risk of fatal CVD based on the European SCORE. However, in women, the prevalence trend of hypertension and the 10-year risk for CVD and fatal CVD decreased significantly, but the prevalence trend of tobacco smoking, high LDL-C and diabetes was stable throughout the six years of screening.

The prevalence of untreated hypertension in our population (population B) is similar to that found in 2006 in the French Nutrition and Health Survey (ENNS)[24]: 14.5% in women (vs 15% ENNS) and 20.3% in men (vs 23.9% ENNS). We observed a significant decrease in the prevalence trend of hypertension in our population of untreated men and women. The prevalence decreased from 23% to 12.7% in women (p< 0.0001) and from 25.9% to 21.1% in men (p = 0.002). Data from the MONICA (MONItoring of trends and determinants in CArdiovascular disease) project [25] and the MONA LISA[26] studies show that the prevalence of hypertension decreased between 1995 and 2005 in France. It was 48% in men and 38% in women in 1995 versus 45% in men and 30% in women in 2005. The decrease is more pronounced in women than in men. More interestingly, the proportion of treated subjects changed very little between 1995 and 2005. Moreover, pooled results from the MONICA project [27] showed similar falls in low, middle, and high readings of blood pressure, implying causes other than hypertensive medication. Therefore, other factors could be responsible for the observed decline in blood pressure.

In our study, we found a significant decrease of prevalence trend for tobacco smoking in men. Legislation banning smoking in public places is increasingly common and is welcomed by the populations concerned. The evidence base is now solid for the effectiveness of smoking bans in CVD prevention. A study by Barone-Adesi [28] indicates that a ban on smoking in all public places was followed 6 months later by a decrease of 11% in admissions for MI in north Italian hospitals. Findings reported by Sargent et al [29] show that emergency admissions for MI decreased by 40% in the state of Montana in the United States during a 6-month ban on smoking in public places and returned to their initial value when the ban was lifted. Bartechi et al [30] reported a 27% decrease in the number of hospital admissions for MI after implementation of the Smoke-Free Air Act in 2003 in Colorado Springs in the United States, while the rate of heart attacks did not change in another city of the same state where there had been no implementation of the anti-smoking law. In our study, the decrease in the prevalence of tobacco smoking was statistically significant in men but not in women. This is consistent with the findings of recent French national surveys.[31, 32]

In our population, the mean 10-year CVD risk decreased from 13.3% to 11.7% in men (p < 0.0001) and from 8.0% to 5.9% in women (p < 0.0001). Women appeared to be at lower CVD risk than men. Ford [33] examined the trends in predicted 10-year risk for CVD from 1999 to 2000 and from 2009 to 2010 among adults in the United States. The mean 10-year CVD risk decreased from 11.8% to 11.5% in men and from 6.6% to 6.2% in women. Favourable trends were noted for mean systolic and diastolic BP and smoking status. In our study, the decline observed in the global risk scores was significant and is consistent with the decline in CV mortality observed in France and the further sharp decline in mortality from cerebrovascular disease.[34]

More recently, the Framingham 10-year CVD risk and the European 10-year risk of fatal CVD were examined in European populations with a 10-year follow-up to determine their validity. Van Dis et al [35] obtained the 10-year follow-up in the EPIC-Netherland cohort. They examined the inclusion of non fatal events in the European 10-year SCORE risk chart and showed that a cut-off point of 10% for total CVD could identify high-risk individuals. Artigao-Rodenas et al [36] showed valid comparisons of prediction models and reality in a random sample of the general population from southern Europe.

Our study is a screening campaign.The main limitation is that we have no follow-up data to present and are therefore not able to assess the possible correction of the detected CVRFs. Another limitation is related to the choice of selected age groups, requiring age adjustment. Our survey was not designed as a longitudinal assessment and re-assessment of CV risk in a community, but rather as a cross-sectional survey spanning several years. It is not known how comparable the parent and sampled populations are across years. There is the possibility of selection bias, and self-selection bias by respondents. Furthermore, no data were available on dietary habits and physical activity, which could have explained some of the observed changes.

Future perspectives should address screening in younger participants and screening at workplaces. Since each French district has a same local health insurance organization, with access codes to the software (http://www.cpam92-si.com/site/frcv/frcv.php), the logistics and the methods used for the screening campaign could be extended to other regions. The estimated 10-year risk for CVD and 10-year risk of fatal CVD should be compared with the observed CV events and the observed CV mortality at 10 years in our population.

In conclusion, over a 6-year period, several CVRFs have decreased in our screening campaign, leading to decrease in the 10-year risk for CVD and the 10-year risk of fatal CVD. Prevention campaign strategies seem efficient and should therefore continue to focus on primary prevention, particularly tobacco control and healthier diets. Cardiologists should recognize the importance of community prevention programs, and communication policies for improving diet and physical activity to decrease the CVRFs in the general population.

## Supporting Information

S1 Table Numbers and demographics of the male and female participants within each year. (DOC)

S2 Table Comparison of the distribution of the cardiovascular risk factors between male and female participants in the untreated participants (population B) and the adjusted model (population C). (DOC)

S3 Table Cardiovascular risk factors and estimated 10-year risk for CVD and fatal CVD in population A (N = 20,324). (DOC)
