# Supplementary material for: Trends in Cardiovascular Disease Risk Factor Prevalence and Estimated 10-Year Cardiovascular Risk Scores in a Large Untreated French Urban Population: The CARVAR 92 Study
Source: PLoS One. 2015 Apr 23;10(4):e0124817. doi: 10.1371/journal.pone.0124817 (PMC4408033; doi:10.1371/journal.pone.0124817)
Supplement: S1 Table — (DOC) [file pone.0124817.s001.doc]

**S1 Table. Numbers and demographics of the male and female participants within each year**

| Men | **2007** | **2008** | **2009** | **2010** | **2011** | **2012** |
| --- | --- | --- | --- | --- | --- | --- |
| N = 9,584 | 1,499 | 2,063 | 1,434 | 1,905 | 1,310 | 1,373 |
| Mean age ± SD | 53.1 ± 4.7 | 55.1 ± 5.3 | 51.3 ± 7.0 | 50.9 ± 6.4 | 45.3 ± 7.1 | 50.9 ± 12.3 |
| Diabetes mellitus (%) | 10.5 | 13.3 | 8.1 | 8.7 | 4.5 | 7.4 |
| Hypertension (%) | 33.7 | 32.0 | 25.2 | 24.0 | 16.5 | 24.3 |
| High LDL cholesterol (%) | 31.6 | 31.4 | 31.9 | 28.5 | 28.7 | 38.7 |
| Current smokers (%) | 34.6 | 21.6 | 27.9 | 27.7 | 25.5 | 23.7 |
| Women | **2007** | **2008** | **2009** | **2010** | **2011** | **2012** |
| N = 10,740 | 1,868 | 1,724 | 1,780 | 1,715 | 2,039 | 1,614 |
| Mean age ± SD | 61.8 ± 4.3 | 63.9 ± 5.0 | 59.4 ± 6.4 | 58.8 ± 6.4 | 53.7 ± 5.6 | 51.2 ± 6.3 |
| Diabetes mellitus (%) | 10.3 | 12.0 | 9.0 | 8.2 | 6.0 | 6.0 |
| Hypertension (%) | 33.7 | 31.8 | 25.1 | 22.3 | 14.2 | 19.6 |
| High LDL cholesterol (%) | 30.0 | 30.0 | 31.4 | 29.9 | 29.7 | 34.0 |
| Current smokers (%) | 24.7 | 16.1 | 20.0 | 21.6 | 20.9 | 21.4 |
