# Supplementary material for: Trends in Cardiovascular Disease Risk Factor Prevalence and Estimated 10-Year Cardiovascular Risk Scores in a Large Untreated French Urban Population: The CARVAR 92 Study
Source: PLoS One. 2015 Apr 23;10(4):e0124817. doi: 10.1371/journal.pone.0124817 (PMC4408033; doi:10.1371/journal.pone.0124817)
Supplement: S2 Table — (DOC) [file pone.0124817.s002.doc]

**S2 Table. Comparison of the distribution of the cardiovascular risk factors between male and female participants in the untreated participants (population B) and the adjusted model (population C).**

| **Men** | **Population B**  **(n=7,401)** | **Population C**  **(n=3,402)** | **P value** |
| --- | --- | --- | --- |
| Diabetes mellitus (%) | 2.6 | 2.2 | 0.23 |
| Hypertension (%) | 20.3 | 20.4 | 0.89 |
| High LDL cholesterol (%) | 24.7 | 24.5 | 0.81 |
| Current smokers (%) | 28.0 | 29.3 | 0.16 |
| Obesity (%) | 11.6 | 12.2 | 0.37 |
| **Women** | **Population B**  **(n=7,308)** | **Population C**  **(n=3,102)** | **P value** |
| Diabetes mellitus (%) | 1.7 | 1.7 | 1 |
| Hypertension (%) | 14.5 | 15.0 | 0.96 |
| High LDL cholesterol (%) | 23.9 | 24.3 | 0.52 |
| Current smokers (%) | 17.0 | 16.9 | 0.66 |
| Obesity (%) | 15.2 | 15.0 | 0.78 |
