# Supplementary material for: Trends in Cardiovascular Disease Risk Factor Prevalence and Estimated 10-Year Cardiovascular Risk Scores in a Large Untreated French Urban Population: The CARVAR 92 Study
Source: PLoS One. 2015 Apr 23;10(4):e0124817. doi: 10.1371/journal.pone.0124817 (PMC4408033; doi:10.1371/journal.pone.0124817)
Supplement: S3 Table — (DOC) [file pone.0124817.s003.doc]

**S3 Table. Cardiovascular risk factors and estimated 10-year risk for CVD and fatal CVD in population A (N=20,324)**

| Men | **2007** | **2008** | **2009** | **2010** | **2011** | **2012** | **P value** |
| --- | --- | --- | --- | --- | --- | --- | --- |
| N=9,584 | 1,499 | 2,063 | 1,434 | 1,905 | 1,310 | 1,373 |  |
| Hypertension (%) | 33.7 | 32.0 | 25.2 | 24.0 | 16.5 | 24.3 | <0.0001 |
| Diabetes mellitus (%) | 10.5 | 13.3 | 8.1 | 8.7 | 4.5 | 7.4 | <0.0001 |
| High LDLc (%) | 31.6 | 31.4 | 31.9 | 28.5 | 28.7 | 38.7 | 0.03 |
| Obesity (%) | 16.9 | 17.7 | 13.7 | 13.2 | 11.6 | 14.2 | <0.0001 |
| Current smokers (%) | 34.6 | 21.6 | 27.9 | 27.7 | 25.5 | 23.7 | 0.0002 |
| 10-year risk for CVD * | 15.9 ± 10.0 | 16.7 ± 10.4 | 14.0 ± 9.8 | 13.4 ± 9.4 | 8.7 ± 7.5 | 14.2 ± 12.9 | <0.0001 |
| 10-year risk of fatal CVD * | 1.3 ± 0.9 | 1.4 ± 1.0 | 1.1 ± 1.0 | 1.0 ± 0.9 | 0.5 ± 0.7 | 1.5 ± 1.9 | <0.0001 |
| Women | **2007** | **2008** | **2009** | **2010** | **2011** | **2012** | **P value** |
| N=10,740 | 1,868 | 1,724 | 1,780 | 1,715 | 2,039 | 1,614 |  |
| Hypertension (%) | 33.7 | 31.8 | 25.1 | 22.3 | 14.2 | 19.6 | <0.0001 |
| Diabetes mellitus (%) | 10.3 | 12.0 | 9.0 | 8.2 | 6.0 | 6.0 | <0.0001 |
| High LDLc (%) | 30.0 | 30.0 | 31.4 | 29.9 | 29.7 | 34.0 | 0.16 |
| Obesity (%) | 24.6 | 21.9 | 20.3 | 21.0 | 18.0 | 19.9 | <0.0001 |
| Current smokers (%) | 24.7 | 16.1 | 20.0 | 21.6 | 20.9 | 21.4 | <0.0001 |
| 10-year risk for CVD * | 10.5 ± 7.0 | 11.3 ± 7.5 | 9.3 ± 6.5 | 8.4 ± 6.1 | 6.1 ± 5.2 | 5.4 ± 4.3 | <0.0001 |
| 10-year risk of fatal CVD * | 1.4 ± 1.0 | 1.7 ± 1.8 | 1.1 ± 1.1 | 1.0 ± 0.9 | 0.5 ± 0.6 | 0.4 ± 0.6 | <0.0001 |

*Mean ± SD

Linear trends were verified using the Cochran-Armitage trend test for linearity for categorical data (diabetes, hypertension, high LDLc, obesity, current smokers), and regression lines for parametric data (10-year risk of fatal CVD and 10-year risk of CVD).

CVD = cardiovascular disease; LDL c = low-density lipoprotein-cholesterol.
